# Development of a YOLOv3-Based Model for Automated Detection of Thoracic Ossification of the Posterior Longitudinal Ligament and the Ligamentum Flavum on Plain Radiographs

**DOI:** 10.3390/jcm14072389

**Published:** 2025-03-31

**Authors:** Sadayuki Ito, Hiroaki Nakashima, Naoki Segi, Jun Ouchida, Ippei Yamauchi, Takashi Hirai, Masahiro Oda, Kensaku Mori, Masashi Yamazaki, Toshitaka Yoshii, Shiro Imagama

**Affiliations:** 1 Department of Orthopaedic Surgery, Nagoya University Graduate School of Medicine, 65 Tsurumaicho, Showa Ward, Nagoya 466-8550, Aichi, Japan; ito.sadayuki.w9@f.mail.nagoya-u.ac.jp (S.I.); naoki.s.n@gmail.com (N.S.); orthochida@gmail.com (J.O.); yaip0411@yahoo.co.jp (I.Y.); imagama.shiro.v4@f.mail.nagoya-u.ac.jp (S.I.); 2 Japanese Multicenter Research Organization for Ossification of the Spinal Ligament, Tokyo 113-8519, Japan; htakacy89@gmail.com (T.H.); masashiy@tsukuba-seikei.jp (M.Y.); yoshii.orth@tmd.ac.jp (T.Y.); 3 Department of Orthopaedic Surgery, Tokyo Medical and Dental University, 1-5-45 Yushima, Bunkyo Ward, Tokyo 113-8519, Japan; 4 Information Strategy Office, Information and Communications, Nagoya University, Nagoya 464-8601, Aichi, Japan; moda@mori.m.is.nagoya-u.ac.jp (M.O.); kensaku@is.nagoya-u.ac.jp (K.M.); 5 Department of Intelligent Systems, Nagoya University Graduate School of Informatics, Nagoya 464-0000, Aichi, Japan; 6 Department of Orthopaedic Surgery, Institute of Medicine, University of Tsukuba, 1-1-1 Tennodai, Tsukuba 305-8575, Ibaraki, Japan

**Keywords:** posterior longitudinal ligament, ligamentum flavum, radiograph, ossification, detection

## Abstract

**Background/Objectives**: This study aims to develop and validate a YOLOv3-based deep learning model for detecting ossification of the posterior longitudinal ligament (OPLL) and ossification of the ligamentum flavum (OLF) on lateral thoracic radiographs, improving early diagnosis and screening accessibility. **Methods**: A retrospective dataset of 356 lateral thoracic radiographs, including 176 with OPLL or OLF and 180 controls, was annotated by spine surgeons. The YOLOv3 model was trained using data augmentation and evaluated via five-fold cross-validation, with accuracy, precision, recall, and F1-score compared to two spine surgeons. **Results**: The model achieved 80.6% accuracy, 70.3% precision, 92.6% recall, and 79.9% F1-score, surpassing spine surgeons in accuracy and recall, especially for combined OPLL and OLF cases. Detection accuracy was 81.1% for OPLL, 53.3% for OLF, and 86.3% for combined cases. **Conclusions**: The YOLOv3-based model provides high accuracy and robust detection of OPLL and OLF on plain radiographs, offering an efficient and accessible screening tool.

## 1. Introduction

Ossification of the posterior longitudinal ligament (OPLL) and ossification of the ligamentum flavum (OLF) are debilitating spinal disorders characterized by abnormal bone formation within the spinal ligaments. These conditions can compress the spinal cord and nerve roots, leading to various neurological complications, including sensory deficits, motor weakness, and, in severe cases, paralysis [1,2]. Several studies have demonstrated that OPLL and OLF significantly impact clinical outcomes, influencing surgical decision-making and patient prognosis. For instance, OPLL has been shown to alter surgical approaches due to its association with increased risk of spinal cord compression, while OLF can severely limit mobility and contribute to chronic pain syndromes [3]. Studies have also reported that OPLL and OLF frequently coexist, affecting different spinal levels and contributing to varying degrees of neurological impairment [4]. OPLL predominantly affects the cervical spine, whereas OLF is more common in the thoracic region, often leading to compressive myelopathy and requiring surgical decompression [5]. Therefore, early detection and intervention are critical to prevent irreversible damage and maintain the quality of life of affected individuals.

OPLL predominantly affects the posterior longitudinal ligament in the cervical and thoracic regions, whereas OLF typically affects the ligamentum flavum in the thoracic and lumbar areas. Notably, both conditions frequently coexist, particularly in aging populations, and are common in East Asia, owing to genetic predisposition and metabolic factors, including diabetes and obesity [6,7,8]. However, despite their clinical significance, timely diagnosis of OPLL and OLF remains challenging, owing to their subtle radiographic features and the overlapping anatomy of the spine. Furthermore, OPLL has been identified as a major challenge in spinal surgery, as its extensive bone formation can complicate minimally invasive approaches such as those utilizing robotic-assisted techniques, including the Da Vinci system, making open surgery a more viable option in many cases [9].

Advanced imaging modalities such as computed tomography (CT) and magnetic resonance imaging (MRI) are highly effective in diagnosing these conditions [10,11]. However, their use for routine screening is limited owing to cost, accessibility, and higher radiation exposure. In contrast, plain radiographs are a more practical first-line diagnostic tool but require extensive expertise to accurately detect early-stage ossification. Therefore, experienced radiologists may face challenges that lead to delayed diagnosis and poorer patient outcomes. Additionally, in cases where OPLL is detected, surgical planning is further complicated by the limited suitability of minimally invasive surgical approaches, particularly robotic-assisted techniques, which may not be feasible for severe ossifications [9].

Thus, to address this challenge, machine and deep learning models have been used to automate the detection of OPLL and other spinal disorders in imaging studies [12,13]. Among the available artificial intelligence (AI) models, the You Only Look Once (YOLO) framework has demonstrated exceptional performance in real-time object detection tasks, including medical imaging. Previous studies utilizing YOLO for spinal disorders have primarily focused on detecting single conditions such as OPLL. OPLL and OLF often coexist, and in clinical practice, models that can detect both OPLL and OLF may be more useful than models that can detect only OPLL; a comprehensive model that can simultaneously identify OPLL and OLF would significantly improve diagnostic efficiency and clinical decision making.

Therefore, in this study, we aimed to develop and validate a modified YOLOv3 model to identify OPLL and OLF on lateral thoracic radiographs. Consequently, by training the model on annotated datasets and comparing its performance with that of experienced spine surgeons, we sought to create an accessible automated diagnostic tool that addresses the limitations of current methods.

## 2. Materials and Methods

### 2.1. Study Population and Data Collection

We conducted this study in compliance with the principles of the Declaration of Helsinki. Our institutional review board approved this study (No. 2016-0177), and the requirement for informed consent was waived owing to its retrospective nature. In this study, we retrospectively analyzed the radiographic and clinical data of patients with thoracic OPLL and OLF to develop a model that could detect both conditions using lateral spinal radiographs. Patients diagnosed with OPLL or OLF based on CT findings and who underwent lateral spine radiography between April 1997 and March 2021 were included in the study. The exclusion criteria included any previous spinal surgery, fractures, or a history of other spinal conditions that could confound diagnostic imaging.

Notably, we used 176 images from patients with OPLL or OLF and 180 images from control participants without spinal ossification, for a total of 356 images. Table 1 shows the baseline characteristics, including age, sex, and vertebral level of ossification. The OPLL and OLF cohorts included 90 men and 86 women, with an average age of 54.9 ± 14.6 years. However, the control cohort comprised 90 men and 90 women, with an average age of 55.7 ± 17.6 years. In the affected cohort, the breakdown of the ossification levels indicated that 112, 97, and 63 cases involved the upper, middle, and lower thoracic spines, respectively. Additionally, the distribution of OPLL types revealed 95 OPLL cases, 30 OLF cases, and 51 OPLL and OLF cases.

### 2.2. Plain Radiograph Dataset

Furthermore, to train and validate the object detection model, we curated a dataset of plain lateral spinal radiographs from the picture archiving and communication system (ours). Only neutral lateral views were selected because they are considered reliable for identifying ossified ligaments. Therefore, the dataset comprised radiographs of patients with and without OPLL or OLF.

### 2.3. Image Preparation and Annotation

**Figure 1:**
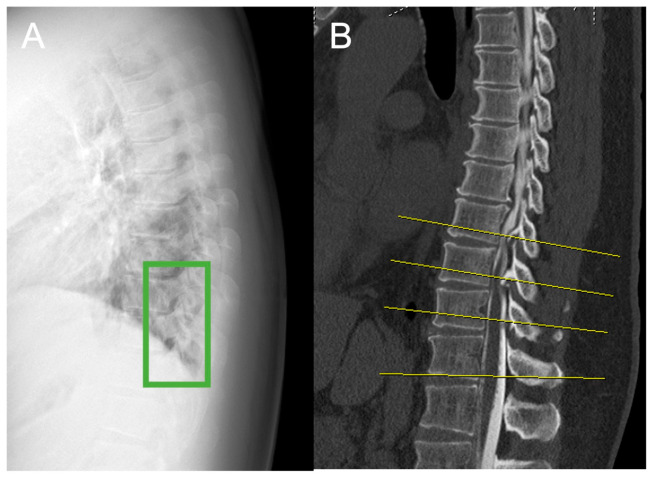
Preparation of images for training the object detection model. Images were annotated with a label by manually inputting a minimal bounding box (a green box) containing the OPLL and OLF on the thoracic lateral radiographs after the exact location of the OPLL was confirmed by CT to generate an image for the object detection training by one orthopedic spine surgeon (15 years): (**A**) dataset image of a plain lateral thoracic spine radiograph; (**B**) sagittal plane CT image to identify OPLL. CT: computed tomography; OPLL: ossification of the posterior longitudinal ligament, OLF: ossification of the ligamentum flavum.

Images were sourced from DICOM files in the hospital’s picture archiving and communication system and exported as JPEG files for use in deep learning. These JPEG images preserve diagnostic details and facilitate efficient processing. An experienced spine surgeon (15 years of experience) annotated each OPLL and OLF lesion by manually placing a bounding box around the affected areas on the lateral radiographs (Figure 1) [14]. This annotation process was verified against CT images to confirm the location and type of ossification (OPLL, OLF, or both).

### 2.4. Object Detection

**Figure 2:**
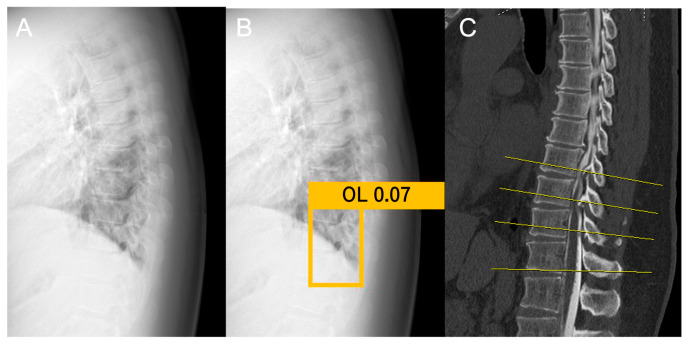
Object detection method. (**A**) plain lateral radiographs of the thoracic spine; (**B**) final region with the highest probability; (**C**) sagittal plane CT image to identify OPLL and OLF. CT: computed tomography; OPLL: ossification of the posterior longitudinal ligament, OLF: ossification of the ligamentum flavum.

In this study, the YOLOv3 model was used for object detection. YOLOv3 was selected owing to its efficiency in real-time object detection, balancing accuracy with faster detection speeds than alternative convolutional neural network models. The model was implemented in Python (version 3.7.7) and trained using the TensorFlow and Keras frameworks. When the model detected an OPLL, a probability (ranging from 0 to 1) was assigned to the detected OPLL. The assigned probabilities were reviewed, and the optimal probability threshold was manually determined through repeated experiments to achieve the best results. The final probability threshold was set at 0.01. All regions with probabilities exceeding the determined threshold were detected. Consequently, multiple regions may be detected, and in such cases, the region with the highest probability was selected (Figure 2).

The training data were randomly categorized into five subsets, with each subset serving as test data once, whereas the remaining data were used for training (five-fold cross-validation). During each folding, 20% of the training subset was reserved as validation data to monitor the learning progress of the model and prevent overfitting. The model was trained on a Quadro P6000 GPU (NVIDIA, Santa Clara, CA, USA) and an Intel Xeon CPU with 64 GB of RAM, employing an Adam optimizer with a learning rate of 0.0001. The optimal detection threshold was determined through iterative testing to maximize the accuracy of the model while minimizing false positives (FPs) and false negatives (FNs). Furthermore, image augmentation, including scaling and horizontal flipping, was applied to enhance training data diversity and improve the robustness of the model.

### 2.5. Performance Evaluation

The performance of the YOLOv3-based object detection model in identifying OPLL and OLF was assessed using multiple metrics to ensure accuracy and reliability. The following metrics were used: accuracy, precision, recall, and F1-score. These metrics are calculated as follows:

Accuracy: The ratio of correctly identified cases (true positives (TPs) and true negatives (TNs)) to the total number of cases.

Precision: This was calculated as the proportion of TP identifications of OPLL and OLF of all cases identified as positive by the model.

Recall: The ratio of true-positive cases correctly identified by the model to the total number of OPLL or OLF cases.

F1-score: The harmonic mean of precision and recall, which provided a balanced measure of model performance (2 × precision × recall/(precision + recall)).

### 2.6. Image Assessment by Doctors

To benchmark the model’s performance against expert-level assessments, two spine surgeons with over 10 years of experience independently reviewed the data. Notably, both surgeons were blinded to the patients’ clinical details to ensure that their evaluations were based solely on imaging data, similar to the input of the model. The surgeons were asked to identify and annotate any regions suspected of having OPLL or OLF on the radiographs. Subsequently, their assessments were compared with the results of the model to evaluate the concordance. Performance metrics were calculated for the surgeons and the model using the same criteria (accuracy, precision, recall, and F1-score) to provide a direct comparison.

### 2.7. Statistical Analysis

The diagnostic peak was determined by calculating the accuracy, precision, recall, and F-measure of the model. The performance of the model was compared with that of two experienced spine surgeons. TPs, FPs, FNs, and TNs were recorded for each detection instance, with statistical analysis conducted using IBM SPSS Statistics for Windows, version 28.0 (IBM Corp., Armonk, NY, USA). The five-fold cross-validation performance metrics were averaged to provide a comprehensive assessment of the model’s generalization ability across various subsets of data.

## 3. Results

### 3.1. Comparative Analysis of True and False Outcomes

To evaluate the performance of the model, the TP, FP, FN, and TN rates were calculated. The model achieved a TP rate of 136/296 (46%) and TN rate of 150/296 (50.7%), with FP and FN rates of 58/296 (19.6%) and 12/296 (4%), respectively (Table 2). Surgeon 1 recorded a TP, TN, FP, and FN rate of 124/296 (41.9%), 156/296 (52.7%), 43/296 (14.5%), and 37/296 (12.5%), respectively. Additionally, Surgeon 2 demonstrated similar TP, TN, FP, and FN rates of 122/296 (41.2%), 144/296 (48.6%), 54/296 (18.2%), and 42/296 (14.2%), respectively.

### 3.2. Overall Model Performance

The YOLOv3-based detection model demonstrated robust performance in identifying OPLL and OLF on lateral thoracic radiographs. The model achieved an overall accuracy of 80.6% with a precision rate, recall, and F1-score of 70.3%, 92.6%, and 79.9%, respectively (Table 3). These metrics indicate the strong capability of the model to recognize true-positive cases while maintaining balanced precision and recall values. Compared with the performance of two experienced spine surgeons, the model showed superior accuracy and recall; however, its precision was slightly lower owing to a higher rate of FPs. Surgeon 1 achieved an accuracy, precision, recall, and F1-score of 78.1%, 74.9%, 77.2%, and 76.0%, respectively. However, surgeon 2 attained an accuracy, precision, recall, and F1-score of 73.8%, 69.5%, 75.0%, and 72.1%, respectively.

### 3.3. Detection Performance Based on Ossification Type

The accuracy of the model varied depending on the type of ossification detected. For OPLL alone, the model achieved an accuracy of 81.1%, indicating its ability to detect distinct ossification patterns in the posterior longitudinal ligament. However, the accuracy for OLF alone was lower (53.3%), indicating challenges in identifying isolated ligamentum flavum ossifications, which are frequently subtle and less visible on plain radiography. The model performed the best in detecting cases involving OPLL and OLF, achieving an accuracy of 86.3%, underscoring its ability to identify more complex cases with overlapping ossification types. Surgeon 1 recorded accuracies of 74.7%, 50.0%, and 76.5% for OPLL, OLF, and combined cases, respectively. However, Surgeon 2 demonstrated accuracies of 72.6%, 53.3%, and 74.5%, respectively (Table 4).

### 3.4. Performance Across Thoracic Spine Levels

The detection accuracy across different thoracic spine levels varied, with the model achieving the highest accuracy in the upper thoracic spine (91.1%), followed by the middle (87.6%) and lower (63.5%) thoracic spines (Table 5). This trend was consistent with the performance of spine surgeons who also demonstrated lower accuracy in the lower thoracic spine. Surgeon 1 achieved accuracies of 84.8%, 78.4%, and 63.5% for the upper, middle, and lower thoracic spine levels, respectively. However, Surgeon 2 achieved accuracies of 83.0%, 75.3%, and 58.7%, respectively. Therefore, high accuracy in the upper thoracic spine may reflect the relatively clear visibility of ossification in this region. Conversely, overlapping anatomical structures in the lower thoracic spine pose challenges for the model and human evaluators.

## 4. Discussion

In this study, we developed an automated detection model using the YOLOv3 deep learning framework to identify thoracic OPLL and OLF on plain radiographs. The model demonstrated promising diagnostic accuracy, comparable to that of experienced spine surgeons, and highlighted the potential of deep learning to assist in the early detection of these conditions. Therefore, by incorporating OPLL and OLF detection within a single framework, this model may contribute to the development of a more efficient diagnostic tool for conditions that often coexist and present challenges for early identification. To the best of our knowledge, this study is the first attempt to integrate T-OPLL and OLF detection within a single AI-driven system using plain radiographs [12,15]. Further validation is necessary; however, the application of YOLOv3 in this context offers an accessible approach, addressing some limitations of advanced imaging modalities, including CT and MRI, which are frequently not feasible for routine screening.

Previous studies implemented YOLOv4 models to detect thoracic OPLL on radiographic images [12,16]. However, in this study, YOLOv3 was selected for its established balance between accuracy and computational efficiency, which is particularly relevant for clinical applications where real-time processing and resource efficiency are essential [17]. The adaptability of YOLOv3 to various object scales and its ability to detect fine details in complex backgrounds make it particularly suitable for thoracic spine imaging, where dense anatomical structures, such as the ribs, vertebrae, and soft tissue, often obscure OPLL and OLF lesions [18]. Moreover, its lighter computational requirements enable its integration into routine clinical workflows, which can accelerate diagnostic processes in settings with limited computational resources.

The performance of the model underscores its adaptability in detecting pathologies in challenging anatomical regions. Therefore, given that thoracic radiographs are frequently more complex than cervical images owing to the overlapping rib and vertebral structures, the precision of YOLOv3 highlights its suitability for addressing these challenges. Compared with CT and MRI, which are frequently used for detailed visualization but have higher costs and additional radiation exposure, the YOLOv3-based model offers a more accessible screening alternative. Therefore, by facilitating accurate initial detection on plain radiographs, this approach enables clinicians to identify patients who may benefit from further diagnostic evaluations such as CT, thereby optimizing the balance between diagnostic accuracy and resource utilization.

Early detection of T-OPLL and OLF is critical because these diseases frequently progress to symptomatic myelopathy if left undiagnosed [19,20]. However, the overlapping anatomies of the thoracic spine make it difficult to identify these diseases using simple radiography. Therefore, OPLL and OLF often go undetected until patients exhibit advanced symptoms, such as severe myelopathy, and the prognosis is generally poor; however, CT and MRI are generally unsuitable for routine screening owing to their high cost and radiation exposure. Therefore, our YOLOv3-based model addresses this gap by providing feasible and low-cost screening using plain radiographs. This enables early intervention and potentially improves patient outcomes.

Our model achieved the highest diagnostic accuracy in cases of combined OPLL and OLF, followed by OPLL- and OLF-only cases. This is especially clinically significant, as combined OPLL and OLF typically represents a more severe pathology with a higher risk of rapid progression, necessitating early intervention to prevent neurological deterioration [11,21,22]. The high accuracy of the model in detecting such complex cases suggests that it could be crucial in supporting timely diagnosis and improving treatment outcomes for patients with high-risk conditions. The detection of OPLL alone was also robust, indicating that the model effectively identified OPLL-specific ossification patterns, which are frequently associated with progressive neurological symptoms if left untreated. However, the lower accuracy in detecting isolated, small OLF lesions highlights a current limitation: subtle and early-stage OLF changes can be challenging to identify owing to their smaller scale and lower visibility on standard imaging. Therefore, addressing this limitation will require further enhancements, potentially through more targeted data augmentation techniques or refined model adjustments, to improve the sensitivity to minor OLF lesions, thereby advancing the model’s overall applicability across various ossification patterns.

Data augmentation is pivotal in optimizing the robustness of a model [23]. Therefore, techniques such as image flipping, rotation, and scaling are employed to enhance the generalizability of the model across a range of imaging conditions. These methods helped address the limitations associated with small dataset sizes, increasing the resilience of the model to variations in image quality, positioning, and patient anatomy. The limited sample size remains a limitation of this study; however, the application of data augmentation serves as a compensatory measure, effectively expanding the diversity of the training data to improve model performance.

AI-based approaches have been increasingly utilized in spinal imaging to improve diagnostic accuracy and efficiency. Recent studies have highlighted the role of machine learning in enhancing the detection and classification of spinal disorders, particularly in complex conditions such as discogenic low back pain (DLBP) and degenerative spinal diseases [24]. The integration of AI models with radiographic imaging has demonstrated the potential to improve early diagnosis and clinical decision-making, reducing dependency on high-cost imaging modalities such as MRI and CT. Furthermore, AI-assisted image analysis can aid in prioritizing high-risk cases, ensuring timely intervention and reducing diagnostic delays. As suggested in recent reviews, combining AI-based detection models with patient-specific data, including neurological assessment scores and biomarkers, may further refine diagnostic accuracy and provide a more comprehensive evaluation of spinal pathologies [25].

The success of our model demonstrates the potential of machine learning tools to provide clinically valuable diagnostic support for spinal imaging, even in regions with dense anatomical overlap. Therefore, by reducing the dependency on more costly imaging techniques, this model contributes to efficient diagnostic pathways and aligns with global efforts toward reducing radiation exposure in patient populations. Machine learning-based diagnostic tools are likely to become the central components of patient care, offering precise and scalable solutions in diagnostic radiology.

## 5. Limitations

This study had some limitations that should be addressed to improve the reliability and applicability of the proposed T-OPLL and OLF detection systems. Despite the promising findings, this study has several limitations. First, the number of radiographs used in the training and validation phases was relatively small, particularly in cases of isolated OLF or less common combinations of ossification patterns. The performance of the model can be enhanced by expanding the dataset to include more varied cases and rare OLF configurations. Data augmentation was applied to diversify the training dataset; however, further data collection from various medical institutions is recommended to improve the generalizability and robustness of the system. Considering the number of X-ray images from past reports, it is thought that about 600–800 images are sufficient to construct a highly accurate model in this study. Therefore, further improvement of accuracy can be expected by adding about 200–400 images [26]. Second, only lateral thoracic radiographs were used in this study. Incorporating frontal images can provide additional spatial information and improve the detection accuracy of OLF, which frequently presents as small or subtle ossification. However, the simplicity of using only lateral images enables rapid analysis and straightforward implementation in clinical settings, with an average processing time of 0.1–0.2 s per image. Furthermore, the model was trained solely on radiographic images without the integration of clinical data such as patient neurological scores (Japanese Orthopaedic Association scores), which could provide critical context for prioritizing high-risk cases. Therefore, adding such clinical information in future iterations of the model could enhance diagnostic accuracy, especially in cases where radiographic findings are less pronounced [27].

## 6. Conclusions

We developed an automated detection model based on the YOLOv3 framework to identify OPLL and OLF on lateral thoracic radiographs. The model showed encouraging results, achieving diagnostic accuracy comparable to that of experienced spinal surgeons. The findings suggest that this model can assist in the early diagnosis of spinal ossification disorders; however, some limitations, including small dataset size and focus on lateral radiographs, should be addressed in future research. Expanding the dataset, incorporating additional imaging modalities, and integrating clinical data may further enhance the reliability and utility of the model. Therefore, we hope that this study will serve as a step toward improving early detection and patient outcomes in these challenging conditions.

## Figures and Tables

**Table 1:** Baseline characteristics of the patients.

		Patients	Controls
N		176	180
Sex (M/F)		90/86	90/90
Age (years)		54.9 ± 14.6	55.7 ± 17.6
Height (cm)		161.3 ± 10.0	158.3 ± 12.1
Weight (kg)		79.9 ± 21.1	59.6 ± 16.2
Level of thoracic spine	Upper	112	n.a.
	Middle	97	n.a.
	Lower	63	n.a.
Type of Ossification	OPLL	95	n.a.
	OLF	30	n.a.
	OPLL+OLF	51	n.a.

Values are presented as mean ± standard deviation for each group. n.a.: not applicable, OPLL: ossification of the posterior longitudinal ligament, OLF: ossification of the ligamentum flavum.

**Table 2:** Diagnostic performance of our detection system and that of spine surgeons 1 and 2.

	Detection (n)			
	TP	FP	FN	TN
Object detection	136	58	12	150
Spine surgeon 1	124	43	37	156
Spine surgeon 2	122	54	42	144

TP, true positive; FP, false positive; FN, false negative; TN, true negative.

**Table 3:** Accuracy of our object detection system and that of spine surgeons 1 and 2.

	AC (%)	PR (%)	RR (%)	F (%)
Object detection	80.6	70.3	92.6	79.9
Spine surgeon 1	78.1	74.9	77.2	76.0
Spine surgeon 2	73.8	69.5	75.0	72.1

AC, accuracy; PR, precision rate; RR, recall rate; F, F-measure.

**Table 4:** Accuracy of our system and that of spine surgeons 1 and 2 for the different types of ossification.

OPLL Type	Accuracy (%)		
	Object Detection	Surgeon 1	Surgeon 2
OPLL	81.1	74.7	72.6
OLF	53.3	50.0	53.3
OPLL+OLF	86.3	76.5	74.5

OPLL: ossification of the posterior longitudinal ligament, OLF: ossification of the ligamentum flavum.

**Table 5:** Accuracy of our system for the upper, middle, and lower thoracic spine.

Level of the Thoracic Spine	Accuracy (%)		
	Object Detection	Surgeon 1	Surgeon 2
Upper	91.1	84.8	83.0
Middle	87.6	78.4	75.3
Lower	63.5	63.5	58.7

## Data Availability

The datasets generated during and analyzed during the current study are not publicly available but are available from the corresponding author on reasonable request.

## Abbreviations

YOLOv3: You Only Look Once version3; OPLL: ossification of the posterior longitudinal ligament; OLF: ossification of the ligamentum flavum; CT: computed tomography; TP, true positive; FP, false positive; FN, false negative; TN, true negative; AC, accuracy; PR, precision rate; RR, recall rate; F, F-measure.
